# Supplementary material for: Empirical methods for controlling false positives and estimating confidence in ChIP-Seq peaks
Source: BMC Bioinformatics. 2008 Dec 5;9:523. doi: 10.1186/1471-2105-9-523 (PMC2628906; doi:10.1186/1471-2105-9-523)
Supplement: Additional File 1 — A variety of html documents from the USeq web site detailing the available applications, their best usage, output file type descriptions, command line menus, etc. [file 1471-2105-9-523-S1.zip › USeqUserGuides/JavaDoc.zip!/JavaDoc/util/gen/IO.html]

IO


|  |  |  |
| --- | --- | --- |
| **Overview** \| **Package** \| **Class** \| **Use** \| **Tree** \| **Deprecated** \| **Index** \| **Help** | |  |
| **PREV CLASS**   **NEXT CLASS** | **FRAMES**    **NO FRAMES**     **All Classes** |  |
| SUMMARY: NESTED \| FIELD \| CONSTR \| METHOD | DETAIL: FIELD \| CONSTR \| METHOD |  |


---


## util.gen Class IO

```
java.lang.Object
  util.gen.IO
```

---

``` public class IO extends java.lang.Object ```

Static methods for Input Output related tasks.

---

**Constructor Summary**

`IO()`


| **Method Summary** | |
| --- | --- |
| `static java.util.LinkedHashMap` | `buildDirectoryFileGroups(java.lang.String map)`             Converts a String of "grp1=/my/Dir1,grp2=/my/Dir2, etc that contains directories into a LinkedHashMap. |
| `static java.util.ArrayList` | `buildFileGroups(java.lang.String map, java.lang.String directoryFileExtensionFilter)`             Converts a String of "grp1=/my/file1,grp1=/my/old/file2,grp2=/my/file2, grp2=/my/new/file3,grp3=/my/new/dir/,/my/default etc" to a File[][] where the files have been broken by grouping into different File[]. |
| `static boolean` | `bunzip2(java.io.File zippedFile, java.io.File decompressedFile)`             Uncompressed bzip2 files using the Ant implementation. |
| `static java.lang.String[]` | `bunZip2(java.io.File bunZip2App, java.io.File bz2FileToUncompress)`             Use to read in xxx.bz2 files directly to a String[]. |
| `static boolean` | `bzip2(java.io.File toZip, java.io.File zipped)`             Uses Ants implementation of the bzip2 compression algorithm. |
| `static void` | `checkFile(java.lang.String par)`             Check to see that a file exists and is not a directory. |
| `static java.io.File[]` | `collapseFileArray(java.io.File[][] f)`             Merges all files in File[][] to a File[]. |
| `static java.lang.String` | `concatinateFileFullPathNames(java.io.File[] f, java.lang.String seperator)`             Concatinates the full path file names for the given File array. |
| `static boolean` | `copy(java.io.File source, java.io.File dest)`             Fast & simple file copy. |
| `static boolean` | `copyDirectoryRecursive(java.io.File sourceDir, java.io.File destDir, java.lang.String extension)`             Copies a given directory and it's contents to the destination directory. |
| `static long` | `countNonBlankLines(java.io.File file)`             Counts the number of lines in a file skipping blanks. |
| `static long` | `countNumberOfLines(java.io.File file)`             Counts the number of lines in a file |
| `static int` | `countNumberThatExist(java.io.File[] f)`             Counts the number of File in the array that actually exist. |
| `static boolean` | `deleteDirectory(java.io.File directory)`             Deletes a directories contents, and then the directory. |
| `static boolean` | `deleteFile(java.io.File file)`             Attempts to delete a file. |
| `static boolean` | `deleteFile(java.lang.String fullPathFileName)`             Attempts to delete a file. |
| `static boolean` | `deleteFiles(java.io.File[] files)`             Attempts to delete the array of File. |
| `static boolean` | `deleteFiles(java.io.File directory, java.lang.String extension)`             Deletes files in a given directory with a given extension. |
| `static void` | `deleteFiles(java.io.File directory, java.lang.String prefix, int minutes)`             Deletes files in a given directory with a given prefix, that are older cutoff. |
| `static void` | `deleteFiles(java.lang.String directory, java.lang.String endsWith)`             Looks in the given directory for files ending with given String and deletes them. |
| `static void` | `deleteFilesNotEndingInExtension(java.io.File directory, java.lang.String extension)`             Deletes files in a given directory that don't end in a given extension. |
| `static java.lang.String[]` | `executeCommandLine(java.lang.String[] command)`             Executes tokenized params on command line, use full paths. |
| `static java.lang.String[]` | `executeCommandLine(java.lang.String[] command, java.io.File txtOutPutFile)`             Executes tokenized params on command line, use full paths. |
| `static java.lang.String[]` | `executeCommandLine(java.lang.String[] command, java.io.File txtOutPutFile, java.lang.String[] env)`             Executes tokenized params on command line, use full paths. |
| `static java.lang.String[]` | `executeCommandLineReturnAll(java.lang.String[] command)`             Executes tokenized params on command line, use full paths. |
| `static java.lang.String[]` | `executeShellScript(java.lang.String shellScript, java.io.File tempDirectory)`             Executes a String of shell script commands via a temp file. |
| `static java.io.File[]` | `extractFiles(java.io.File directory)`             Extracts the full path file names of all the files and directories in a given directory. |
| `static java.io.File[]` | `extractFiles(java.io.File dirOrFile, java.lang.String extension)`             Extracts the full path file names of all the files in a given directory with a given extension (ie txt or .txt). |
| `static java.io.File[]` | `extractFiles(java.lang.String commaSeparList)`             Extracts all files from a comma delimited string of files and directories. |
| `static java.io.File[]` | `extractFiles(java.lang.String[] dirFiles, java.lang.String extension)`             Given full path file or directory names, extracts all files given an extension. |
| `static java.io.File[]` | `extractFiles(java.lang.String commaSeparList, java.lang.String extension)`             Extracts all files with a given extension from a comma delimited string of files and directories. |
| `static java.io.File[]` | `extractFilesReturnFiles(java.io.File directory, java.lang.String extension)`             Extracts the full path file names of all the files in a given directory with a given extension. |
| `static java.lang.String[]` | `extractFilesStringNames(java.io.File directory, java.lang.String extension)`             Extracts the full path file names of all the files in a given directory with a given extension. |
| `static java.io.File[]` | `extractOnlyDirectories(java.io.File directory)`             Returns directories or null if none found. |
| `static java.io.File[]` | `extractOnlyFiles(java.io.File directory)`             Extracts the full path file names of all the files not directories in a given directory. |
| `static java.util.ArrayList<java.io.File>` | `fetchAllFilesRecursively(java.io.File directory)`             Fetches files that don't start with a '.' from a directory recursing through sub directories. |
| `static java.util.ArrayList` | `fetchAllFilesRecursively(java.io.File directory, java.lang.String extension)`             Fetches all files with a given extension in a directory recursing through sub directories. |
| `static java.util.ArrayList` | `fetchArrayList(java.io.File file)`             Fetches an ArrayList stored as a serialize object file. |
| `static java.lang.String[]` | `fetchFileNames(java.io.File[] files)`             Returns the names of the files or directories. |
| `static java.io.File[]` | `fetchFiles(java.lang.String[] baseNames, java.lang.String chromExtension)`             For each base name (ie /home/data/treat1 ), makes a File object using the chromExtenstion (ie /home/data/treat1.chromExtension ), if it actually exists, it is saved and a File[] returned. |
| `static java.io.File[]` | `fetchFilesRecursively(java.io.File directory)`             Fetches all files in a directory recursing through sub directories. |
| `static java.io.File[]` | `fetchFilesRecursively(java.io.File directory, java.lang.String extension)`             Fetches all files with a given extension in a directory recursing through sub directories. |
| `static float[][][]` | `fetchFloatFloatArrays(java.io.File[] files)`             Loads float[][]s from disk. |
| `static java.util.HashMap` | `fetchNamesAndDirectories(java.io.File directory)`             Given a directory, returns a HashMap of the containing directories' names and a File obj for the directory. |
| `static java.lang.Object` | `fetchObject(java.io.File file)`             Fetches an Object stored as a serialized file. |
| `static java.io.BufferedReader` | `fetchReaderOnZippedFile(java.io.File zippedFile)`             Returns a BufferedReader from which you can call readLine() directly from a zipped file without decompressing. |
| `static java.lang.String` | `fireQSub(java.lang.String qsubParams, java.lang.String commands, java.io.File tempDir)`             Fires qsub with the commands. |
| `static java.lang.String[]` | `fireQSub(java.lang.String queue, java.lang.String commands, java.lang.String fullPathToTempDir, boolean waitAndClean)`             Fires qsub with the commands, will wait and return results and delete x.sh and the output files if you like. |
| `static java.lang.String` | `getFullPathName(java.io.File fileDirectory)`             Gets full path name using getCanonicalPath() on a File |
| `static java.lang.String[]` | `getTruncatedNames(java.io.File[] files)`             Fetches and truncates common chars, from file names, front and back, uses getName() so no path info is involved. |
| `static java.lang.String[][]` | `getTruncatedNames(java.io.File[][] files)`             Fetches and truncates common chars, from file names, front and back, uses getName() so no path info is involved. |
| `static java.lang.String[]` | `loadFile(java.io.File file)`             Loads a file's lines into a String[], will save blank lines. |
| `static java.util.HashMap` | `loadFileIntoHashMap(java.io.File file)`             Loads a file's lines into a hash first column is the key, second the value. |
| `static java.util.HashSet<java.lang.String>` | `loadFileIntoHashSet(java.io.File file)`             Loads a file's lines into a hash set, keys only. |
| `static java.util.HashSet` | `loadFileIntoHashSplitLines(java.io.File file)`             Loads a file's lines into a hash. |
| `static java.util.LinkedHashSet` | `loadFileIntoLinkedHashSet(java.io.File file)`             Loads a file's lines into a linked hash set, keys only. |
| `static java.lang.String[]` | `loadFileIntoStringArray(java.io.File file)`             Loads a file's lines into a String[] trimming blank lines and beginning and ending whitespace. |
| `static java.util.LinkedHashMap` | `loadHash(java.io.File tabDelimited)`             Loads a tab delimited file into a LinkedHashMap where each line is broken into cells and loaded into a LinkedHashMap where the cells are the keys and the value is the line. |
| `static java.util.LinkedHashMap` | `loadKeyValueFile(java.io.File file)`             Loads a key = value containing text file into a LinkedHashMap. |
| `static java.util.LinkedHashMap` | `loadKeyValueSpaceTabDelimited(java.io.File file, boolean printErrorMessages)`             Loads a key value containing text file into a LinkedHashMap. |
| `static int[][]` | `loadTableOfInts(java.io.File file)`             Loads a tab delimited file containing ints into an int[columns][rows], all lines included, assumes no missing values. |
| `static java.lang.String` | `makeFullPathName(java.lang.String resultsDirectory, java.lang.String fileName)`             Attempts to make a file and return it's canonical path name (full path name). |
| `static java.util.HashMap` | `makeNameFileHash(java.io.File[] files)`             Returns a HashMap of the file name sans extension and the file. |
| `static int` | `numberFilesExist(java.io.File[] f)`             Returns the number of files that exist in the array. |
| `static int` | `numberFilesExist(java.io.File directory, java.lang.String extension)`             Returns the number of files that end with the given extension. |
| `static java.lang.String[]` | `parseColumn(java.io.File file, int index)`             Parses the indexed column of a tab delimited file, all lines included. |
| `static java.util.HashMap` | `parseFile(java.io.File file, int index, boolean ignoreDuplicateKeys)`             Parses a tab delimited file, the indexed column is used as the key, the entire line as the value, blank lines skipped, returns null if a duplicate key is found. |
| `static void` | `removeExtension(java.io.File directory, java.lang.String extension)`             Removes the extension from each File in the directory. |
| `static void` | `renameFiles(java.io.File directory, java.lang.String toReplace, java.lang.String replaceWith)`             Renames files containing the toReplace with replaceWith using String.replaceAll(String, String) |
| `static boolean` | `saveObject(java.io.File file, java.lang.Object ob)` |
| `static java.lang.String` | `winFileConvert(java.lang.String fileName, javax.servlet.http.HttpServletRequest request)`             Checks to see if the browser sending the request is from a windows machine. |
| `static boolean` | `writeArrayList(java.util.ArrayList al, java.io.File file)`             Writes each element of an Arraylist on a seperate line to a file. |
| `static boolean` | `writeHashMap(java.util.HashMap hashMap, java.io.File file)`             Writes each key tab value on a separate line to the file. |
| `static boolean` | `writeString(java.lang.String data, java.io.File file)`             Writes a String to disk. |
| `static boolean` | `writeString(java.lang.String data, java.lang.String fullPathFileName)`             Writes a String to disk. |
| `static boolean` | `writeStringAppend(java.lang.String data, java.io.File file)`             Writes a String onto the end of a file. |
| `static boolean` | `zip(java.io.File[] filesToZip, java.io.File zipFile)`             Zip compresses an array of Files, be sure to name your zipFile with a .zip extension! |
| `static boolean` | `zip(java.io.File fileToZip, java.io.File zipFile)`             Zip compress a single file. |
| `static boolean` | `zipAndDelete(java.io.File f)`             Zip compresses the file and if sucessful, then deletes the original file. |
| `static java.lang.String[]` | `zippedFile2StringArray(java.io.File zippedFile)`             Returns the contents of a zipped file. |

**Methods inherited from class java.lang.Object**

`equals, getClass, hashCode, notify, notifyAll, toString, wait, wait, wait`

**Constructor Detail**

### IO

```
public IO()
```


**Method Detail**

### fetchFileNames

```
public static java.lang.String[] fetchFileNames(java.io.File[] files)
```

:   Returns the names of the files or directories.

---


### bzip2

```
public static boolean bzip2(java.io.File toZip,
                            java.io.File zipped)
```

:   Uses Ants implementation of the bzip2 compression algorithm.
    Be sure to name your file xxx.bz2

---


### bunzip2

```
public static boolean bunzip2(java.io.File zippedFile,
                              java.io.File decompressedFile)
```

:   Uncompressed bzip2 files using the Ant implementation.
    Be sure to name your file xxx.bz2

---


### buildDirectoryFileGroups

```
public static java.util.LinkedHashMap buildDirectoryFileGroups(java.lang.String map)
```

:   Converts a String of "grp1=/my/Dir1,grp2=/my/Dir2, etc that contains
    directories into a LinkedHashMap.
    Returns null and prints an error message if an error was thrown, ie no directory, not a directory

---


### buildFileGroups

```
public static java.util.ArrayList buildFileGroups(java.lang.String map,
                                                  java.lang.String directoryFileExtensionFilter)
```

:   Converts a String of "grp1=/my/file1,grp1=/my/old/file2,grp2=/my/file2,
    grp2=/my/new/file3,grp3=/my/new/dir/,/my/default etc"
    to a File[][] where the files have been broken by grouping into different File[].
    If no key is provided, the files are assigned to a common 'default' grouping.
    If a directory is provided all files within are grouped together.
    Mixing of keyed and unkeyed is permitted.
    Returns null if an error was thrown.

    :   **Parameters:**: `directoryFileExtensionFilter` - - if directories are given in the map, you can filter which files to extract (ie 'cel'), case insensitive, set to "." for everything. **Returns:**: - ArrayList containing String[] groupNames and a File[][] of grouped files, and a LinkedHashMap of the two.

---


### bunZip2

```
public static java.lang.String[] bunZip2(java.io.File bunZip2App,
                                         java.io.File bz2FileToUncompress)
```

:   Use to read in xxx.bz2 files directly to a String[].
    Returns null if a problem encountered.
    Must have bunzip2 installed.

---


### checkFile

```
public static void checkFile(java.lang.String par)
```

:   Check to see that a file exists and is not a directory.

---


### collapseFileArray

```
public static java.io.File[] collapseFileArray(java.io.File[][] f)
```

:   Merges all files in File[][] to a File[].

---


### concatinateFileFullPathNames

```
public static java.lang.String concatinateFileFullPathNames(java.io.File[] f,
                                                            java.lang.String seperator)
```

:   Concatinates the full path file names for the given File array.

---


### copy

```
public static boolean copy(java.io.File source,
                           java.io.File dest)
```

:   Fast & simple file copy. From GForman http://www.experts-exchange.com/M\_500026.html

---


### copyDirectoryRecursive

```
public static boolean copyDirectoryRecursive(java.io.File sourceDir,
                                             java.io.File destDir,
                                             java.lang.String extension)
```

:   Copies a given directory and it's contents to the destination directory.
    Use a extension (e.g. "class$|properties$") to limit the files copied over or set to null for all.

---


### countNonBlankLines

```
public static long countNonBlankLines(java.io.File file)
```

:   Counts the number of lines in a file skipping blanks.

---


### countNumberOfLines

```
public static long countNumberOfLines(java.io.File file)
```

:   Counts the number of lines in a file

---


### countNumberThatExist

```
public static int countNumberThatExist(java.io.File[] f)
```

:   Counts the number of File in the array that actually exist.

---


### deleteDirectory

```
public static boolean deleteDirectory(java.io.File directory)
```

:   Deletes a directories contents, and then the directory.
    Returns false if at any time a file cannot be deleted.

---


### deleteFile

```
public static boolean deleteFile(java.io.File file)
```

:   Attempts to delete a file.

---


### deleteFile

```
public static boolean deleteFile(java.lang.String fullPathFileName)
```

:   Attempts to delete a file.

---


### deleteFiles

```
public static boolean deleteFiles(java.io.File directory,
                                  java.lang.String extension)
```

:   Deletes files in a given directory with a given extension.

---


### deleteFiles

```
public static void deleteFiles(java.io.File directory,
                               java.lang.String prefix,
                               int minutes)
```

:   Deletes files in a given directory with a given prefix, that are older cutoff.

---


### deleteFiles

```
public static boolean deleteFiles(java.io.File[] files)
```

:   Attempts to delete the array of File.

---


### deleteFiles

```
public static void deleteFiles(java.lang.String directory,
                               java.lang.String endsWith)
```

:   Looks in the given directory for files ending with given String and deletes them.

---


### deleteFilesNotEndingInExtension

```
public static void deleteFilesNotEndingInExtension(java.io.File directory,
                                                   java.lang.String extension)
```

:   Deletes files in a given directory that don't end in a given extension.

---


### executeCommandLine

```
public static java.lang.String[] executeCommandLine(java.lang.String[] command)
```

:   Executes tokenized params on command line, use full paths.
    Put each param in its own String.
    Returns null if a problem is encountered.

---


### executeCommandLine

```
public static java.lang.String[] executeCommandLine(java.lang.String[] command,
                                                    java.io.File txtOutPutFile)
```

:   Executes tokenized params on command line, use full paths.
    Put each param in its own String.
    Writes lines to a file.

---


### executeCommandLine

```
public static java.lang.String[] executeCommandLine(java.lang.String[] command,
                                                    java.io.File txtOutPutFile,
                                                    java.lang.String[] env)
```

:   Executes tokenized params on command line, use full paths.
    Put each param in its own String.
    Writes lines to a file.

---


### executeCommandLineReturnAll

```
public static java.lang.String[] executeCommandLineReturnAll(java.lang.String[] command)
```

:   Executes tokenized params on command line, use full paths.
    Put each param in its own String.
    Returns null if a problem is encountered.
    Returns both error and data from execution.

---


### executeShellScript

```
public static java.lang.String[] executeShellScript(java.lang.String shellScript,
                                                    java.io.File tempDirectory)
```

:   Executes a String of shell script commands via a temp file. Only good for Unix.

---


### extractFiles

```
public static java.io.File[] extractFiles(java.io.File directory)
```

:   Extracts the full path file names of all the files and directories in a given directory. If a file is given it is
    returned as the File[0].
    Skips files starting with a '.'

---


### extractOnlyDirectories

```
public static java.io.File[] extractOnlyDirectories(java.io.File directory)
```

:   Returns directories or null if none found. Not recursive.
    Skips those beginning with a period.

---


### extractFiles

```
public static java.io.File[] extractFiles(java.io.File dirOrFile,
                                          java.lang.String extension)
```

:   Extracts the full path file names of all the files in a given directory with a given extension (ie txt or .txt).
    If the dirFile is a file and ends with the extension then it returns a File[] with File[0] the
    given directory. Returns null if nothing found. Case insensitive.

---


### extractFiles

```
public static java.io.File[] extractFiles(java.lang.String commaSeparList)
```

:   Extracts all files from a comma delimited string of files and directories.
    No spaces. Returns null if file.canRead() is false. Not recursive.

---


### extractFiles

```
public static java.io.File[] extractFiles(java.lang.String commaSeparList,
                                          java.lang.String extension)
```

:   Extracts all files with a given extension from a comma delimited string of files and directories.
    No spaces.

---


### extractFiles

```
public static java.io.File[] extractFiles(java.lang.String[] dirFiles,
                                          java.lang.String extension)
```

:   Given full path file or directory names, extracts all files given an extension.

---


### extractFilesReturnFiles

```
public static java.io.File[] extractFilesReturnFiles(java.io.File directory,
                                                     java.lang.String extension)
```

:   Extracts the full path file names of all the files in a given directory with a given extension.

---


### extractFilesStringNames

```
public static java.lang.String[] extractFilesStringNames(java.io.File directory,
                                                         java.lang.String extension)
```

:   Extracts the full path file names of all the files in a given directory with a given extension.

---


### extractOnlyFiles

```
public static java.io.File[] extractOnlyFiles(java.io.File directory)
```

:   Extracts the full path file names of all the files not directories in a given directory.
    Skips files starting with a non word character (e.g. '.', '\_', etc).
    Returns null if no files found.

---


### fetchAllFilesRecursively

```
public static java.util.ArrayList<java.io.File> fetchAllFilesRecursively(java.io.File directory)
```

:   Fetches files that don't start with a '.' from a directory recursing through sub directories.

---


### fetchAllFilesRecursively

```
public static java.util.ArrayList fetchAllFilesRecursively(java.io.File directory,
                                                           java.lang.String extension)
```

:   Fetches all files with a given extension in a directory recursing through sub directories.

---


### fetchArrayList

```
public static java.util.ArrayList fetchArrayList(java.io.File file)
```

:   Fetches an ArrayList stored as a serialize object file.

---


### fetchFiles

```
public static java.io.File[] fetchFiles(java.lang.String[] baseNames,
                                        java.lang.String chromExtension)
```

:   For each base name (ie /home/data/treat1 ), makes a File object using the chromExtenstion
    (ie /home/data/treat1.chromExtension ), if it actually exists, it is saved and a File[]
    returned.

---


### fetchFilesRecursively

```
public static java.io.File[] fetchFilesRecursively(java.io.File directory)
```

:   Fetches all files in a directory recursing through sub directories.

---


### fetchFilesRecursively

```
public static java.io.File[] fetchFilesRecursively(java.io.File directory,
                                                   java.lang.String extension)
```

:   Fetches all files with a given extension in a directory recursing through sub directories.
    Will return a file if a file is given with the appropriate extension, or null.

---


### fetchFloatFloatArrays

```
public static float[][][] fetchFloatFloatArrays(java.io.File[] files)
```

:   Loads float[][]s from disk.

---


### fetchNamesAndDirectories

```
public static java.util.HashMap fetchNamesAndDirectories(java.io.File directory)
```

:   Given a directory, returns a HashMap of the containing directories' names and a File obj for the directory.

---


### fetchObject

```
public static java.lang.Object fetchObject(java.io.File file)
```

:   Fetches an Object stored as a serialized file.
    Can be zip compressed too.

---


### fetchReaderOnZippedFile

```
public static java.io.BufferedReader fetchReaderOnZippedFile(java.io.File zippedFile)
```

:   Returns a BufferedReader from which you can call readLine() directly from a zipped file without decompressing.
    Returns null if there is a problem.

---


### fireQSub

```
public static java.lang.String fireQSub(java.lang.String qsubParams,
                                        java.lang.String commands,
                                        java.io.File tempDir)
```

:   Fires qsub with the commands.

    :   **Parameters:**: `qsubParams` - = '-q queue@loc -l nodes=1' **Returns:**: String beginning with 'Error: ' if the error stream or java bombed. otherwise it returns a tab delimited list of the output and the qsub parameters. Will leave the shell file in the temp directory, renamed as the jobNumber.sh

---


### fireQSub

```
public static java.lang.String[] fireQSub(java.lang.String queue,
                                          java.lang.String commands,
                                          java.lang.String fullPathToTempDir,
                                          boolean waitAndClean)
```

:   Fires qsub with the commands, will wait and return results and delete x.sh and the output files if you like.
    Be sure to put an end of line '\n' after each command.

---


### getFullPathName

```
public static java.lang.String getFullPathName(java.io.File fileDirectory)
```

:   Gets full path name using getCanonicalPath() on a File

---


### getTruncatedNames

```
public static java.lang.String[] getTruncatedNames(java.io.File[] files)
```

:   Fetches and truncates common chars, from file names, front and back, uses getName() so no path info is involved.

---


### getTruncatedNames

```
public static java.lang.String[][] getTruncatedNames(java.io.File[][] files)
```

:   Fetches and truncates common chars, from file names, front and back, uses getName() so no path info is involved.

---


### loadFile

```
public static java.lang.String[] loadFile(java.io.File file)
```

:   Loads a file's lines into a String[], will save blank lines.

---


### loadFileIntoHashMap

```
public static java.util.HashMap loadFileIntoHashMap(java.io.File file)
```

:   Loads a file's lines into a hash first column is the key, second the value.

---


### loadFileIntoHashSet

```
public static java.util.HashSet<java.lang.String> loadFileIntoHashSet(java.io.File file)
```

:   Loads a file's lines into a hash set, keys only.

---


### loadFileIntoHashSplitLines

```
public static java.util.HashSet loadFileIntoHashSplitLines(java.io.File file)
```

:   Loads a file's lines into a hash.

---


### loadFileIntoLinkedHashSet

```
public static java.util.LinkedHashSet loadFileIntoLinkedHashSet(java.io.File file)
```

:   Loads a file's lines into a linked hash set, keys only.

---


### loadFileIntoStringArray

```
public static java.lang.String[] loadFileIntoStringArray(java.io.File file)
```

:   Loads a file's lines into a String[] trimming blank lines and beginning and ending whitespace.

---


### loadHash

```
public static java.util.LinkedHashMap loadHash(java.io.File tabDelimited)
```

:   Loads a tab delimited file into a LinkedHashMap where each line is broken into cells and loaded into
    a LinkedHashMap where the cells are the keys and the value is the line.

---


### loadKeyValueFile

```
public static java.util.LinkedHashMap loadKeyValueFile(java.io.File file)
```

:   Loads a key = value containing text file into a LinkedHashMap.
    Strips all white space, ignores lines beginning with / or #.
    Ignores blank lines.

---


### loadKeyValueSpaceTabDelimited

```
public static java.util.LinkedHashMap loadKeyValueSpaceTabDelimited(java.io.File file,
                                                                    boolean printErrorMessages)
```

:   Loads a key value containing text file into a LinkedHashMap.
    Ignores lines beginning with / or #.
    Ignores blank lines.
    Splits on spaces/ tabs thus no spaces within key or value.
    Returns null if split keyValue number is != 2.

---


### loadTableOfInts

```
public static int[][] loadTableOfInts(java.io.File file)
```

:   Loads a tab delimited file containing ints into an int[columns][rows], all lines included, assumes no missing values.

---


### makeFullPathName

```
public static java.lang.String makeFullPathName(java.lang.String resultsDirectory,
                                                java.lang.String fileName)
```

:   Attempts to make a file and return it's canonical path name (full path name).

---


### makeNameFileHash

```
public static java.util.HashMap makeNameFileHash(java.io.File[] files)
```

:   Returns a HashMap of the file name sans extension and the file.
    Returns null if duplicate name is found.

---


### numberFilesExist

```
public static int numberFilesExist(java.io.File directory,
                                   java.lang.String extension)
```

:   Returns the number of files that end with the given extension.

---


### numberFilesExist

```
public static int numberFilesExist(java.io.File[] f)
```

:   Returns the number of files that exist in the array.

---


### parseColumn

```
public static java.lang.String[] parseColumn(java.io.File file,
                                             int index)
```

:   Parses the indexed column of a tab delimited file, all lines included.
    Will return an empty String if index column doesn't exist

---


### parseFile

```
public static java.util.HashMap parseFile(java.io.File file,
                                          int index,
                                          boolean ignoreDuplicateKeys)
```

:   Parses a tab delimited file, the indexed column is used as the key,
    the entire line as the value, blank lines skipped, returns null if
    a duplicate key is found.

---


### removeExtension

```
public static void removeExtension(java.io.File directory,
                                   java.lang.String extension)
```

:   Removes the extension from each File in the directory.

---


### renameFiles

```
public static void renameFiles(java.io.File directory,
                               java.lang.String toReplace,
                               java.lang.String replaceWith)
```

:   Renames files containing the toReplace with replaceWith using String.replaceAll(String, String)

---


### saveObject

```
public static boolean saveObject(java.io.File file,
                                 java.lang.Object ob)
```

---


### writeArrayList

```
public static boolean writeArrayList(java.util.ArrayList al,
                                     java.io.File file)
```

:   Writes each element of an Arraylist on a seperate line to a file.

---


### writeHashMap

```
public static boolean writeHashMap(java.util.HashMap hashMap,
                                   java.io.File file)
```

:   Writes each key tab value on a separate line to the file.

---


### winFileConvert

```
public static java.lang.String winFileConvert(java.lang.String fileName,
                                              javax.servlet.http.HttpServletRequest request)
```

:   Checks to see if the browser sending the request is from a windows machine.
    If so it converts the file name dividers to unix.
    In either case, replaces any spaces with underscores.

---


### writeString

```
public static boolean writeString(java.lang.String data,
                                  java.io.File file)
```

:   Writes a String to disk.

---


### writeString

```
public static boolean writeString(java.lang.String data,
                                  java.lang.String fullPathFileName)
```

:   Writes a String to disk.

---


### writeStringAppend

```
public static boolean writeStringAppend(java.lang.String data,
                                        java.io.File file)
```

:   Writes a String onto the end of a file.
    No need to add a \n onto the final end.

---


### zip

```
public static boolean zip(java.io.File fileToZip,
                          java.io.File zipFile)
```

:   Zip compress a single file.

---


### zip

```
public static boolean zip(java.io.File[] filesToZip,
                          java.io.File zipFile)
```

:   Zip compresses an array of Files, be sure to name your zipFile with a .zip extension!

---


### zipAndDelete

```
public static boolean zipAndDelete(java.io.File f)
```

:   Zip compresses the file and if sucessful, then deletes the original file.

---


### zippedFile2StringArray

```
public static java.lang.String[] zippedFile2StringArray(java.io.File zippedFile)
```

:   Returns the contents of a zipped file. Returns null if there is a problem.


---


|  |  |  |
| --- | --- | --- |
| **Overview** \| **Package** \| **Class** \| **Use** \| **Tree** \| **Deprecated** \| **Index** \| **Help** | |  |
| **PREV CLASS**   **NEXT CLASS** | **FRAMES**    **NO FRAMES**     **All Classes** |  |
| SUMMARY: NESTED \| FIELD \| CONSTR \| METHOD | DETAIL: FIELD \| CONSTR \| METHOD |  |


---
